# Supplementary material for: Genome re-sequencing reveals the evolutionary history of peach fruit edibility
Source: Nat Commun. 2018 Dec 20;9:5404. doi: 10.1038/s41467-018-07744-3 (PMC6302090; doi:10.1038/s41467-018-07744-3)
Supplement: Supplementary file 1 — Supplementary Information [file 41467_2018_7744_MOESM1_ESM.pdf]

Genome re-sequencing reveals the evolutionary history of peach fruit edibility

Yu et al.

Supplementary Figures

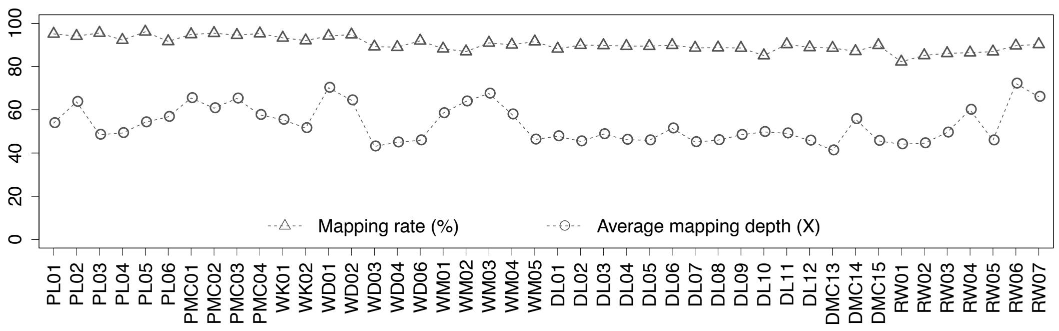

Supplementary Figure 1 The distribution of the mapping rate and final effective mapping depth for each accession. For each accession, the mapping rate ranged from 82.24% to 96.11% and the final effective mapping depth ranged from 41.25× to 72.18×.

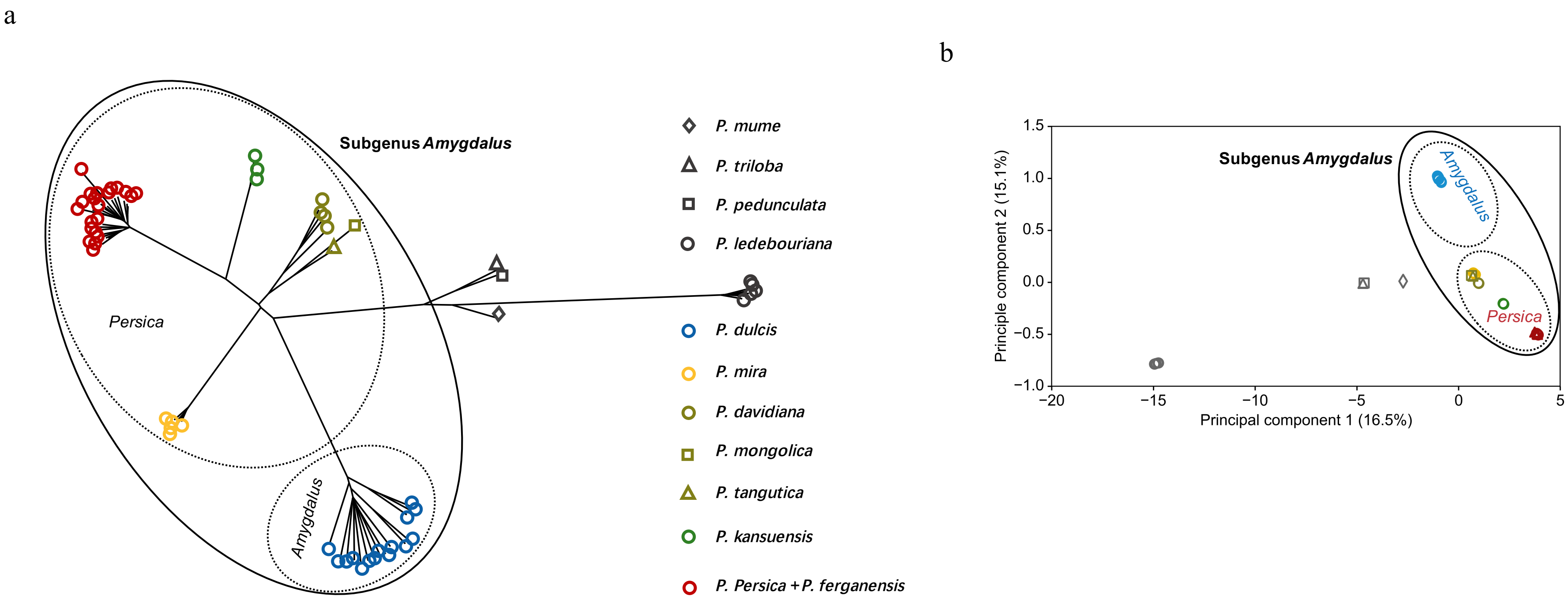

Supplementary Figure 2 A neighbor-joining (NJ) tree analysis (**a**) and principal component analysis (PCA) (**b**) of 58 cultivated peaches and their closely related relatives, as well as *P. mume* (belonging to the genus *Prunus* subgenus *Prunus*) based on 3,909,617 whole-genome SNPs (MAF > 10%, missing rates ≤ 5%). The length of branches indicates simple matching distance. The NJ tree clearly excluded *P. ledebouriana*, *P. triloba*, and *P. pedunculata,* as well as *P. mume,* from subg. *Amygdalus*, and classified *P. tangutica* and *P. mongolica* into the *Persica* section of subg. *Amygdalus*. The pattern was further supported by PCA.

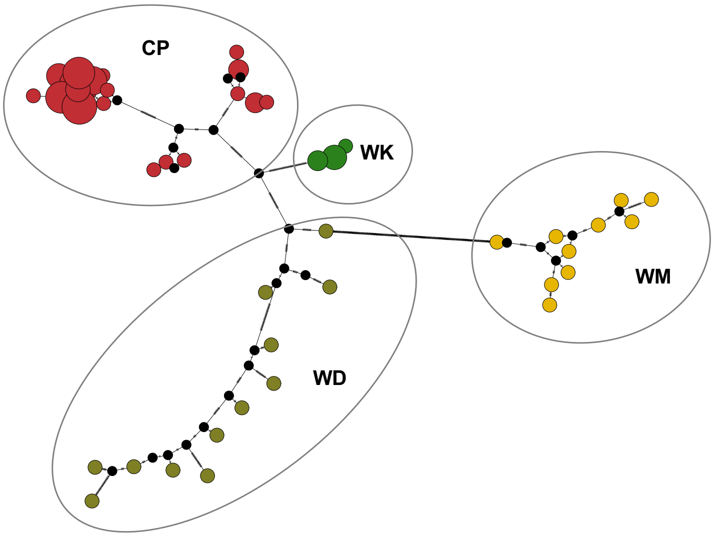

Supplementary Figure 3 Median joint network analysis based on a large haplotype (Pp08, 13449595–13547914; size: 98.32 kb) shared among cultivated and wild relative peaches. Nodes representing haplotypes are colored according to the defined WM, WD, WK, and CP groupings of accessions. Node size and parallel lines on branches are shown in proportion to haplotype frequency and to the extent of mutations, respectively.

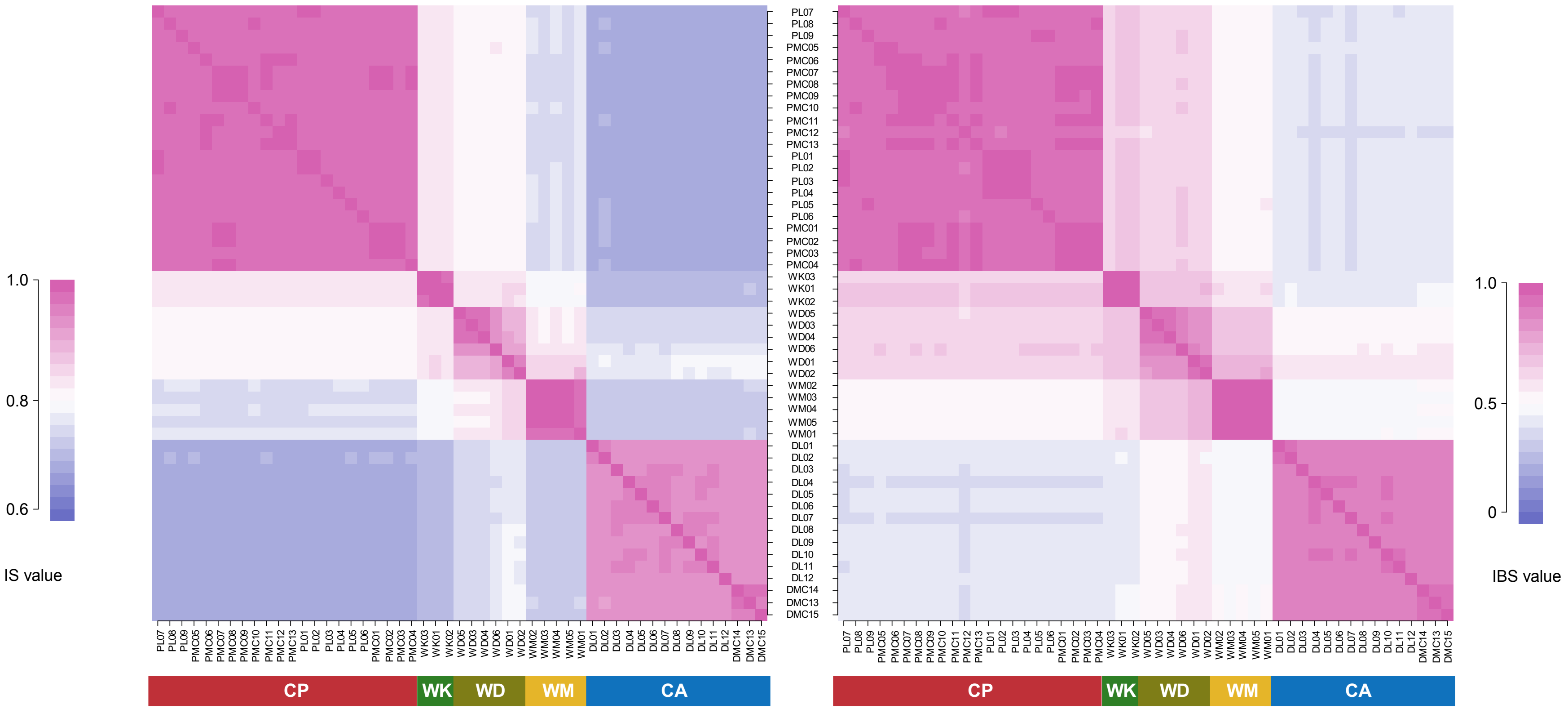

Supplementary Figure 4 Identity score (IS) and identity-by-state (IBS) analyses for the 51 *Amygdalus* accessions. The IS and IBS values of each pair accessions in the same grouping were much higher than those of the pairs in other groupings.

a

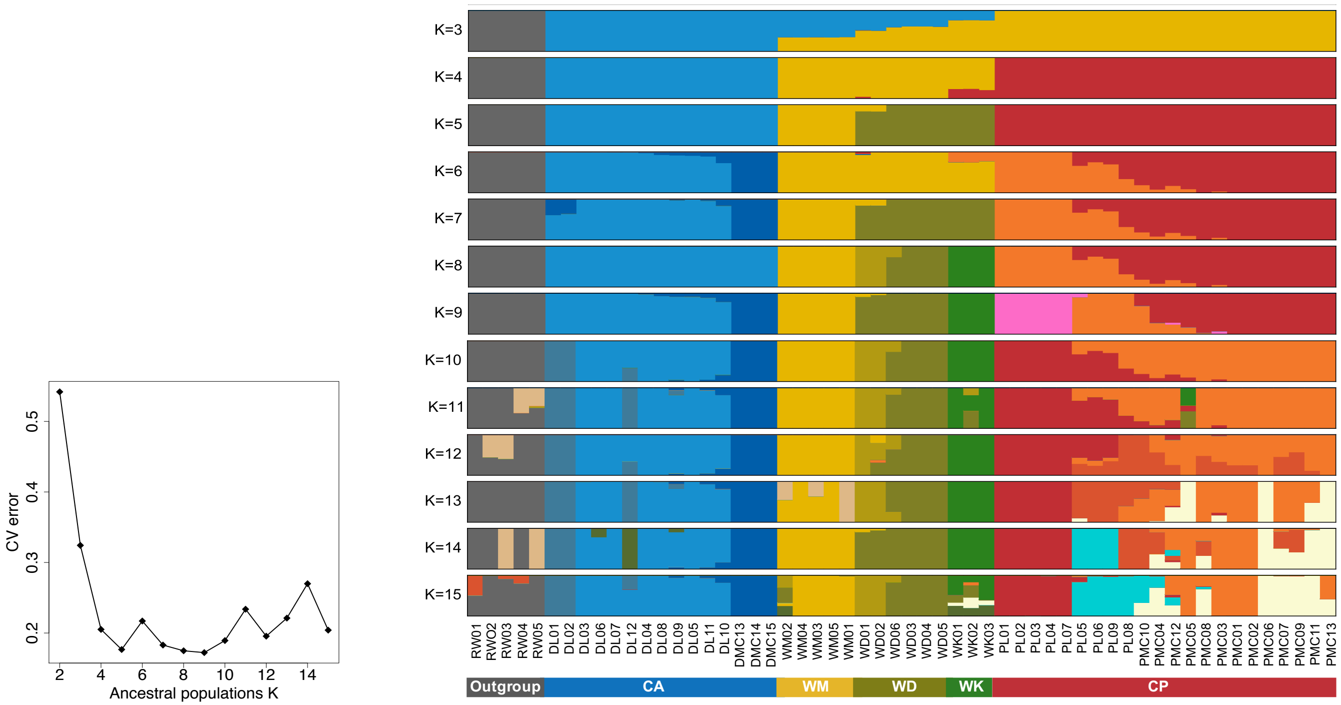
CV error
0.2
0.3
0.4
0.5
2
4
6
8
10
12
14
Ancestral populations K
K=3
K=4
K=5
K=6
K=7
K=8
K=9
K=10
K=11
K=12
K=13
K=14
K=15
RW01 RWO2 RW03 RW04 RW05 DL01 DL02 DL03 DL06 DL07 DL12 DL04 DL08 DL09 DL05 DL11 DL10 DMC13 DMC14 DMC15 WM02 WM04 WM03 WM05 WM01 WD01 WD02 WD06 WD03 WD04 WD05 WK01 WK02 WK03 PL01 PL02 PL03 PL04 PL07 PL05 PL06 PL09 PL08 PMC10 PMC04 PMC12 PMC05 PMC08 PMC03 PMC01 PMC02 PMC06 PMC07 PMC09 PMC11 PMC13
Outgroup
CA
WM
WD
WK
CP

b

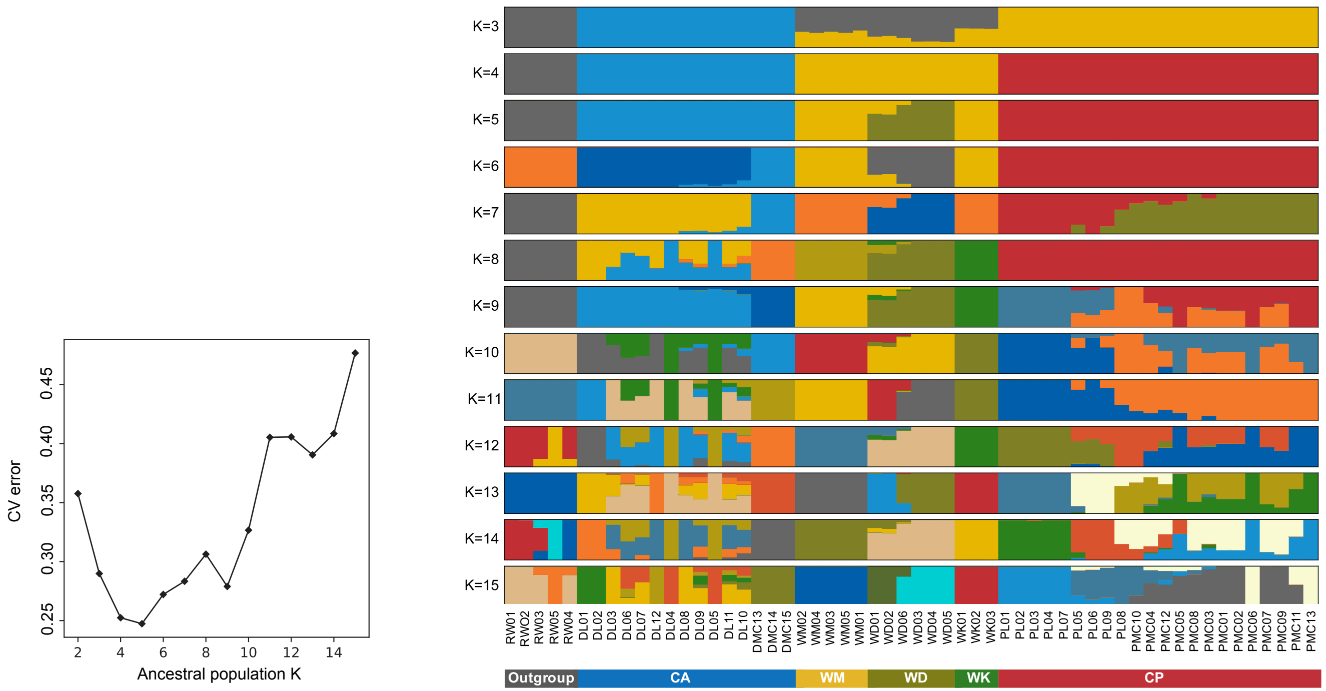

Supplementary Figure 5 Model-based clustering analyses for the 51 *Amygdalus* accessions using *P. ledebouriana* as the outgroup. (**a**) Cross validation (CV) error under different ancestral populations, and the model-based clustering (K = 3 to15) are shown. A unique grouping containing all the *P. ferganensis* accessions (marked with pink color) (K = 9) that were clustered with the *P. persica* landraces from north China (K = 8) was identified. (**b**) We further performed this analysis with filtered SNPs by testing HWE violations ($P > 10^{-4}$); depicted is the CV error and the model-based clustering result (K = 3 to15). This result (K = 9) further supported the grouping patterns that we had identified based on the NJ tree; the *P. ferganensis* accessions were clustered with the *P. persica* landraces from north China (K = 7).

a

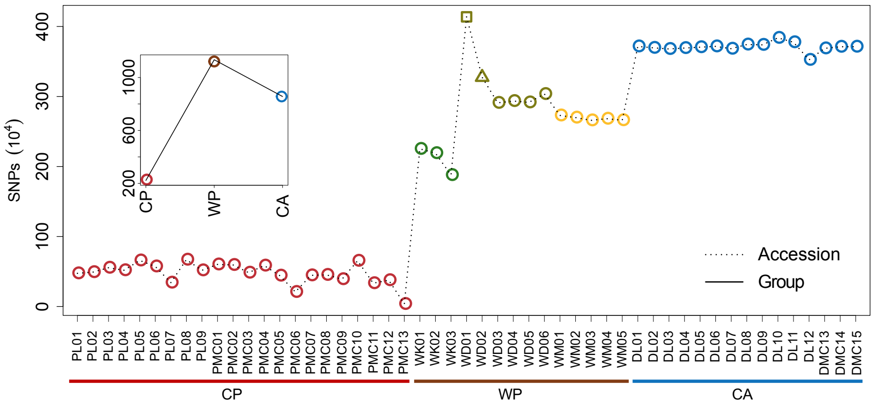

b

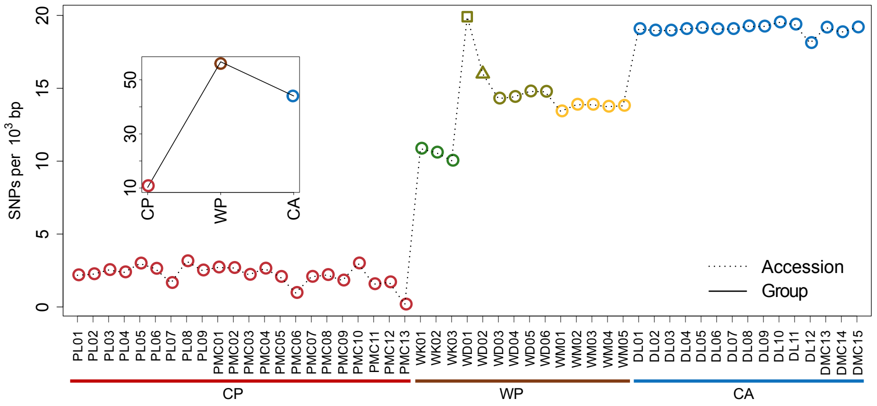

c

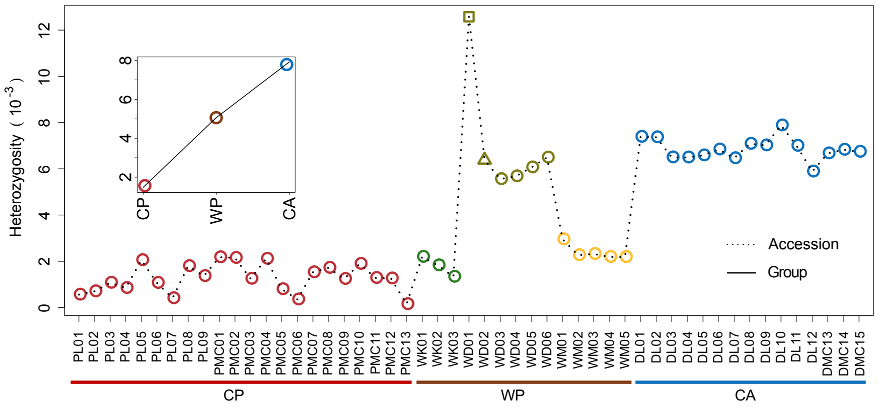

Supplementary Figure 6 Number, frequency and heterozygosity statistics of SNPs for each accession in cultivated peaches (CP), wild relative peaches (WP = WM + WD + WK), and cultivated almonds (CA), and their group levels. (**a**) Number of SNPs; (**b**) Frequency of SNPs per kb; **(c)** Heterozygosity.

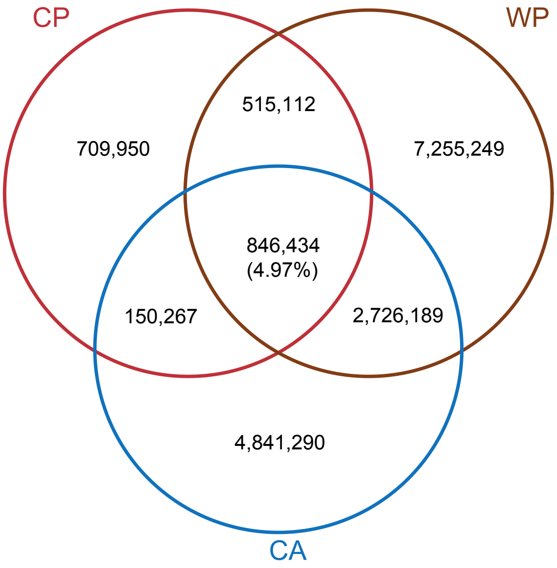

Supplementary Figure 7 Venn diagrams showing the number of common and unique SNPs in the CP, WP, and CA groups. Fewer than ~5% of SNPs were common to all the three groups.

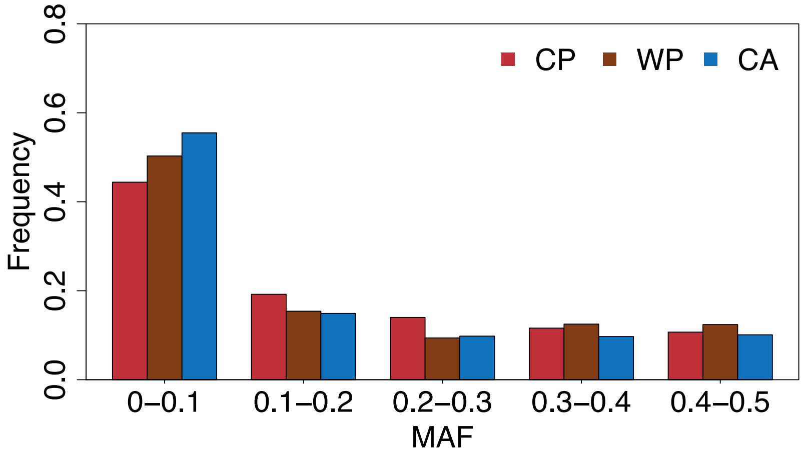

Supplementary Figure 8 Minor allele frequency (MAF) distributions of SNPs in cultivated peaches (CP), wild relative peaches (WP) and cultivated almonds (CA). The proportion of MAF <0.1 was the lowest in cultivated peaches.

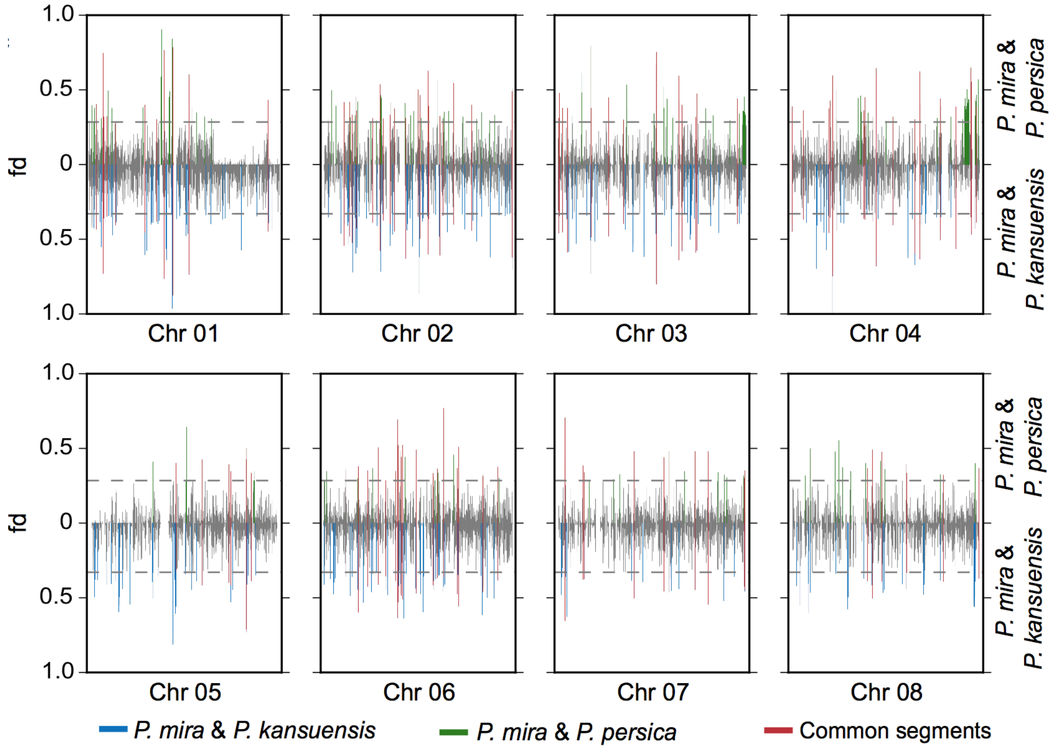

Supplementary Figure 9 Manhattan plots of introgressed segments across all eight chromosomes. Introgressed signatures with significantly positive *D* statistic values (20 kb sliding windows, 5% empirical distribution) between *P. mira* and *P. kansuensis*, and between *P. mira* and *P. persica* are colored in blue and green, respectively. Common introgressed segments with significantly positive *D* statistic values are colored in red.

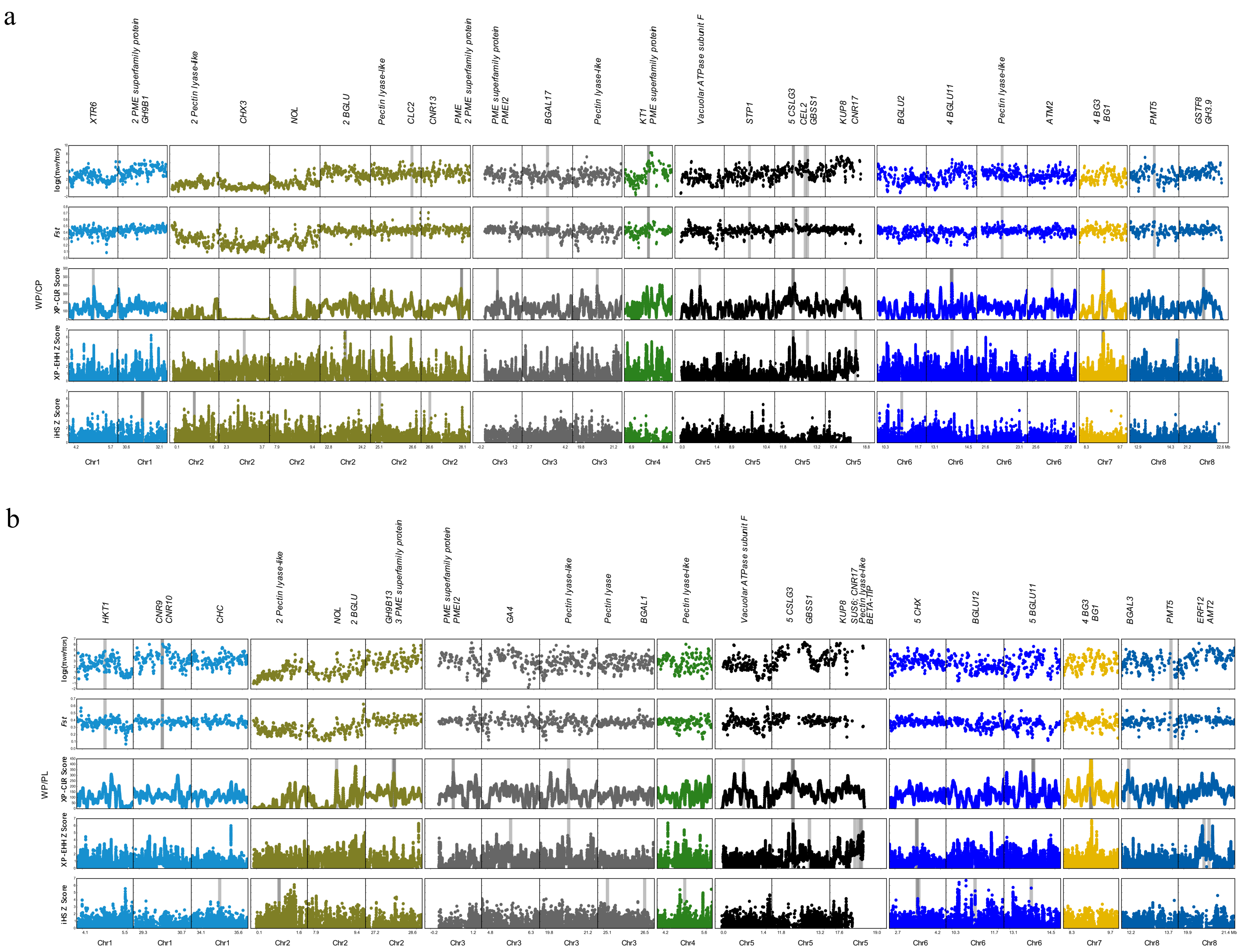

Supplementary Figure 10 Genome-wide selection regions and candidate genes involved in fruit edibility. Positive selection signatures in a comparison of the CP group and the WP group (**a**), and in comparison of PL subgroup and WP group (**b**) are showed in panels presenting distinct selection metrics including F_{ST} & $\theta\pi$ ratio, XP-CLR, XP-EHH, and iHS, respectively. Fruit edibility associated candidate genes are visualized.

a

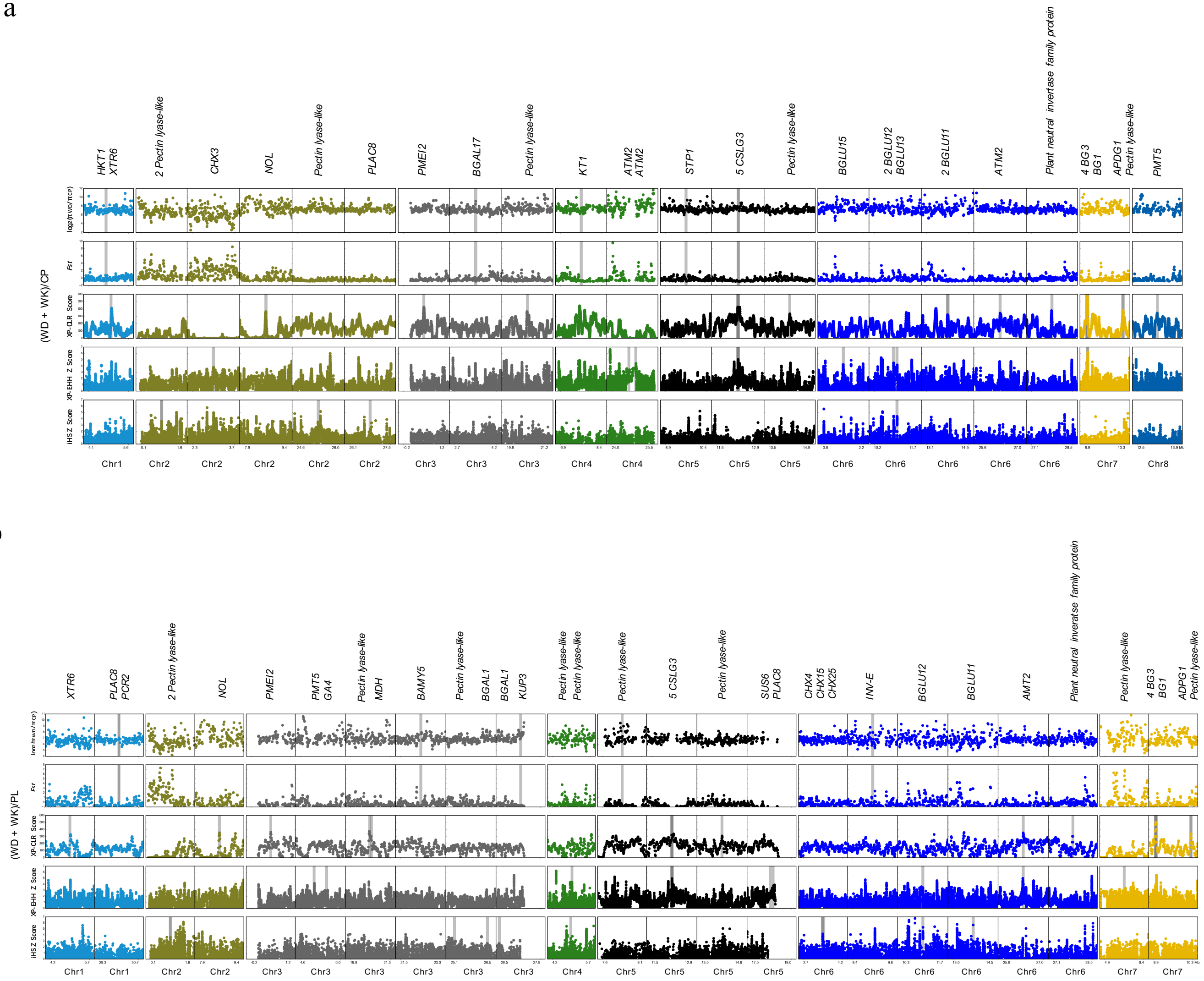

b

Supplementary Figure 11 Genome-wide selection regions and candidate genes involved in fruit edibility. Positive selection signatures in a comparison of the CP group and the (WD + WK) group (**a**), and in comparison of PL subgroup and (WD + WK) group (**b**) are showed in panels presenting distinct selection metrics including F_{ST} & $\theta\pi$ ratio, XP-CLR, XP-EHH, and iHS, respectively. Fruit edibility associated candidate genes are visualized.

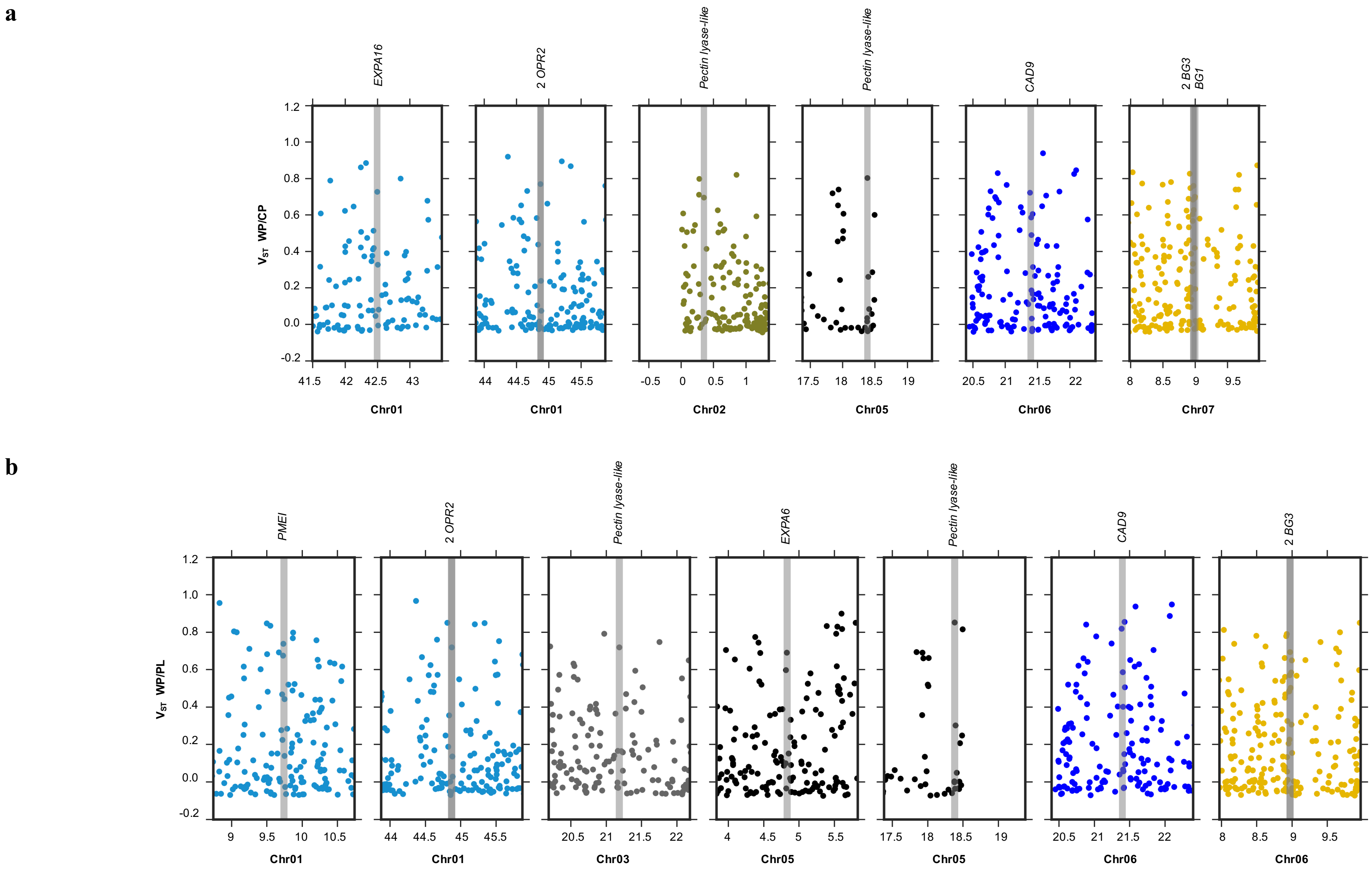

Supplementary Figure 12 Genome-wide selected copy number variations (CNVs) related to fruit edibility. Manhattan plots of V_{ST} values for positive selection signatures in a comparison of the CP group and the WP group (**a**), and in a comparison of PL subgroup and the WP group (**b**). Fruit edibility associated candidate genes are visualized with shadows.

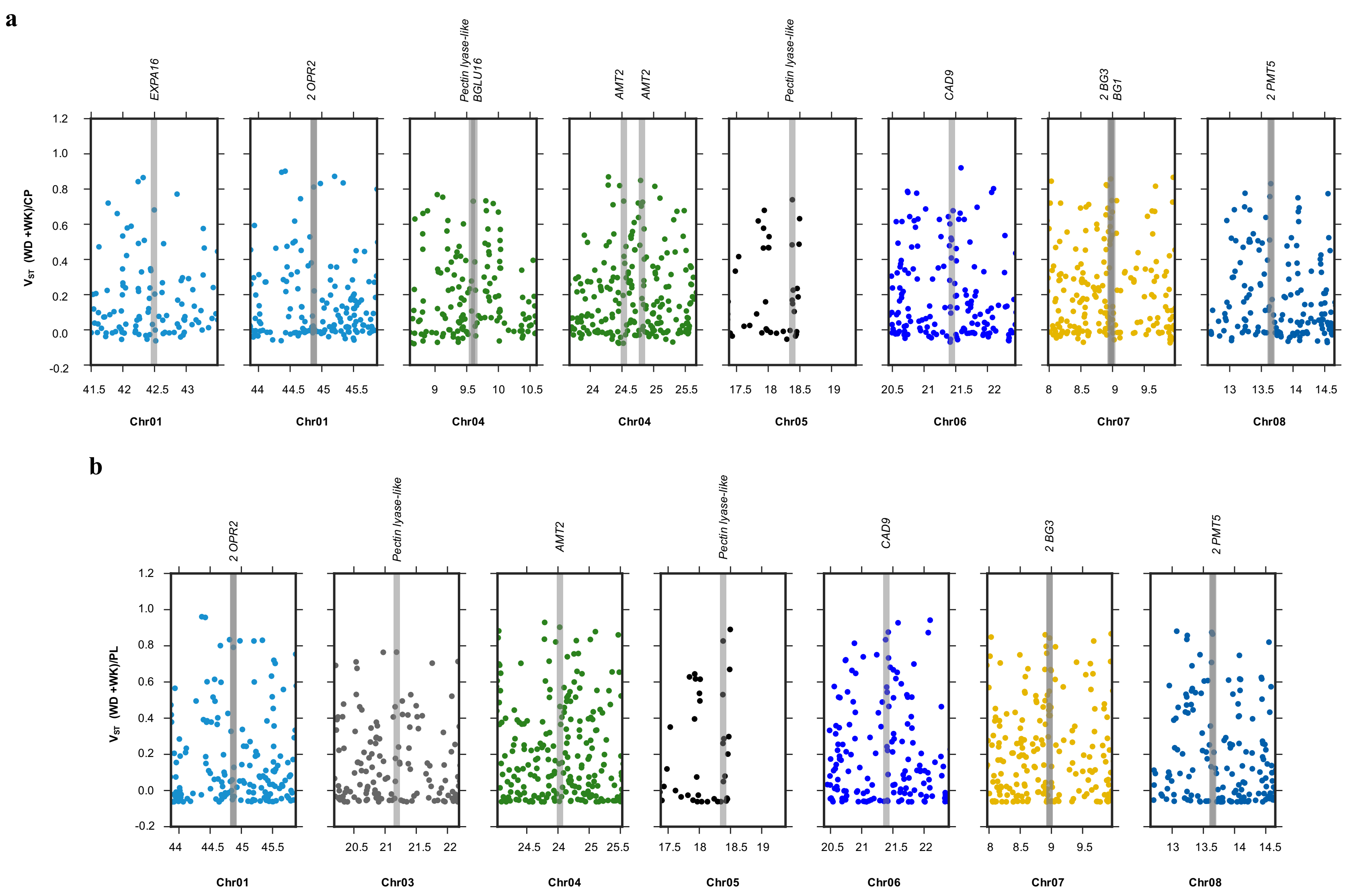

Supplementary Figure 13 Genome-wide selected copy number variations (CNVs) related to fruit edibility. Manhattan plots of V_{ST} values for positive selection signatures in a comparison of the CP group and the (WD + WK) group (**a**), and in a comparison of PL subgroup and the (WD + WK) group (**b**). Fruit edibility associated candidate genes are visualized with shadows.

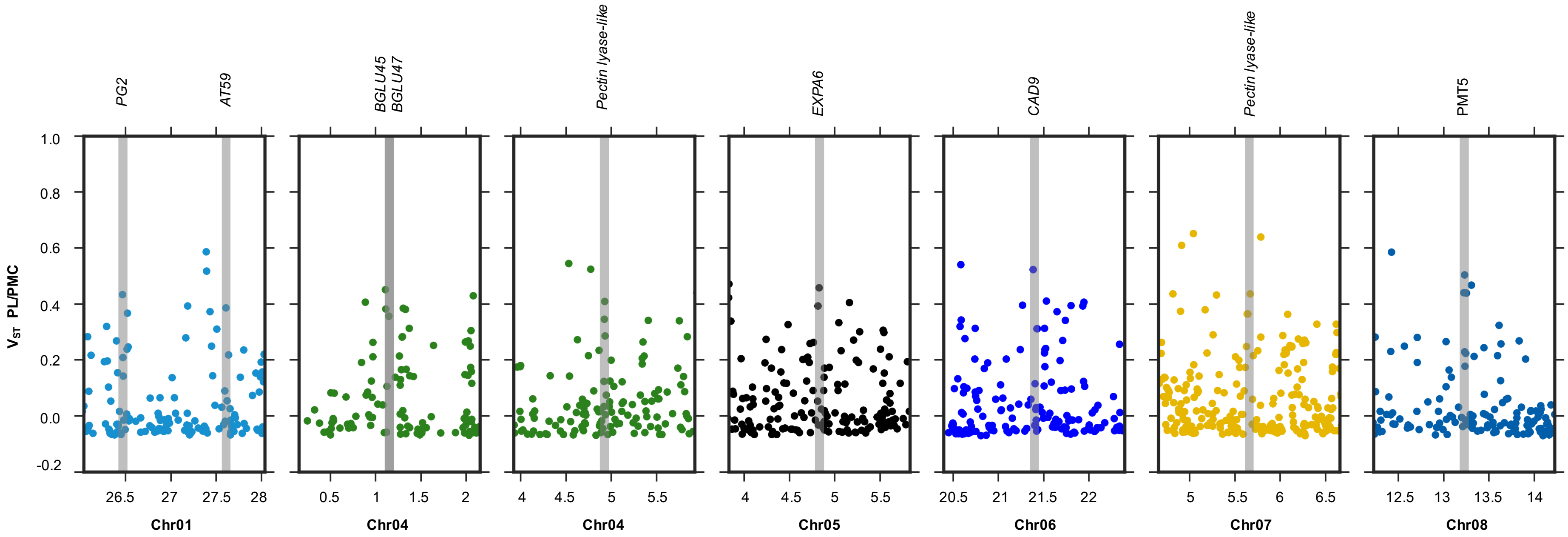

Supplementary Figure 14 Genome-wide selected copy number variations (CNVs) related to fruit edibility. Manhattan plots of V_{ST} values showing positive selection signatures in a comparison of the PL subgroup and the PMC subgroup. Fruit edibility associated candidate genes are visualized with shadows.

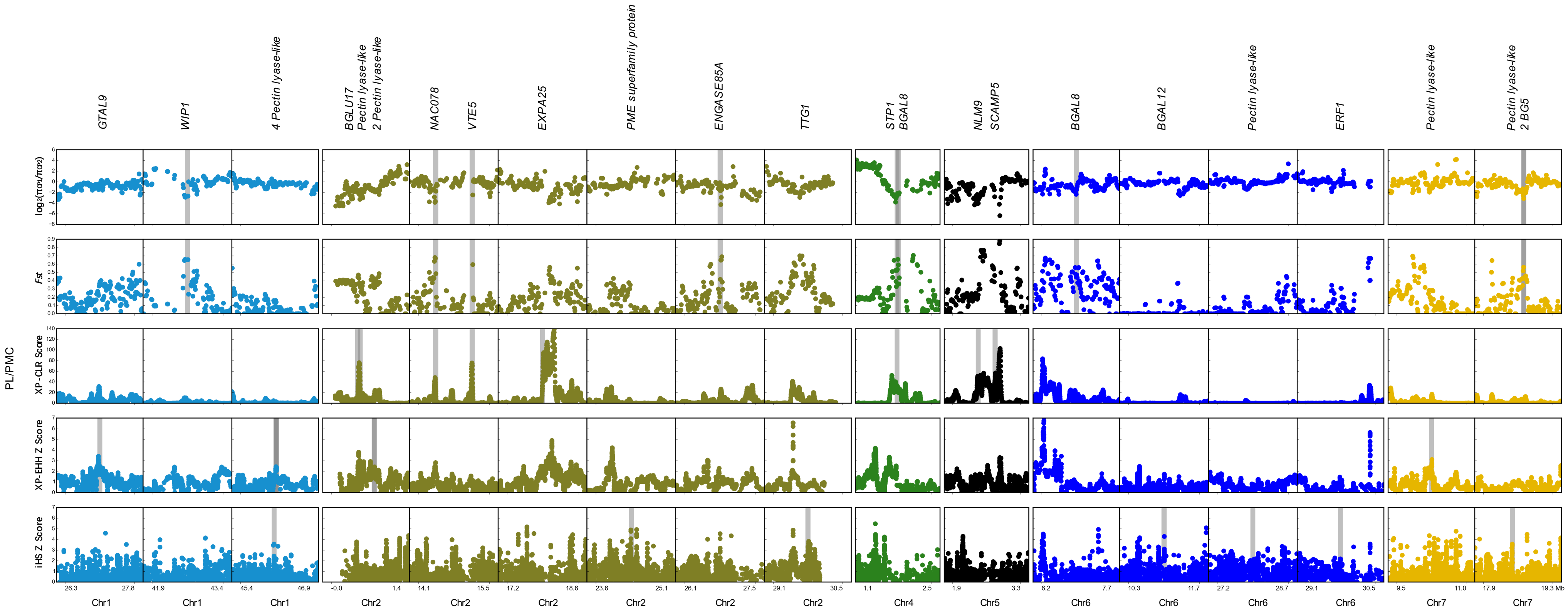

Supplementary Figure 15 Genome-wide selection regions and candidate genes involved in fruit edibility. Positive selection signatures in comparison of the PL subgroup and the PMC subgroup are shown in separate panels presenting distinct selection metrics including F_{ST} & $\theta\pi$ ratio, XP-CLR, XP-EHH, and iHS. Fruit edibility associated candidate genes are visualized.

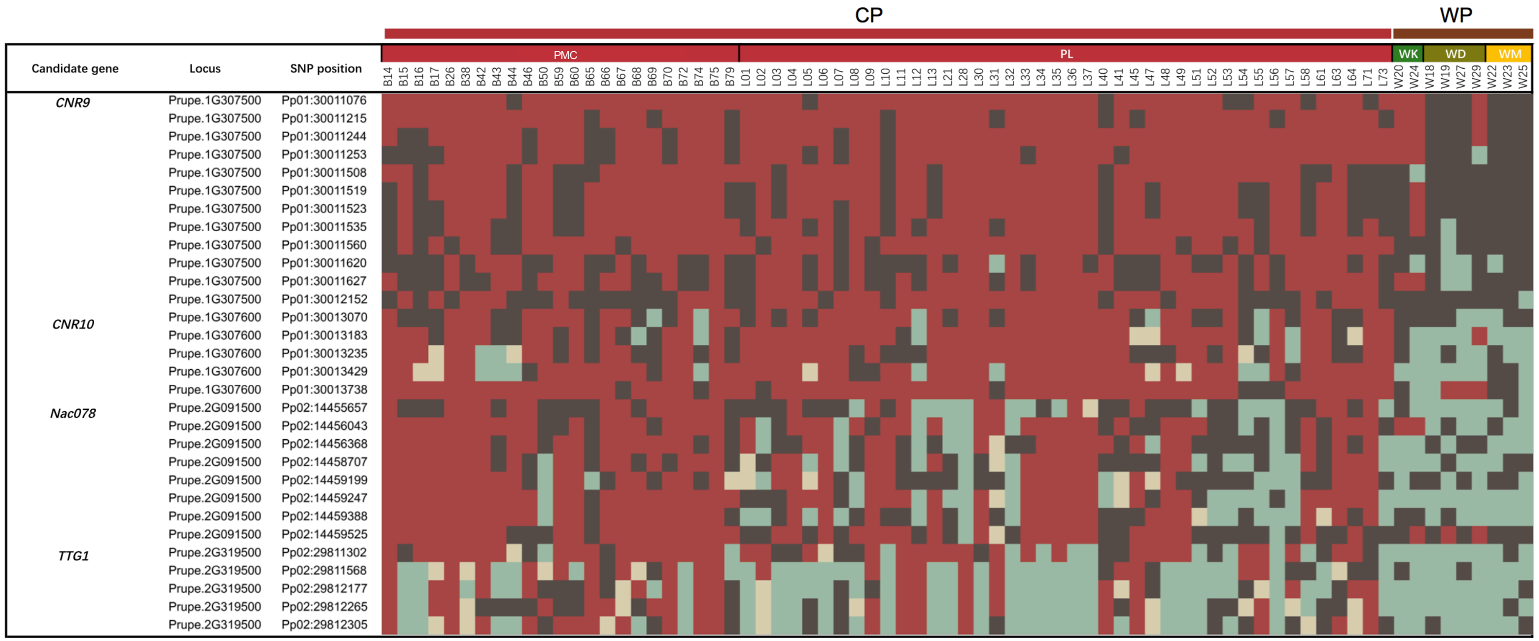

Supplementary Figure 16 Haplotype differentiation of *CNR* genes (*CNR9* and *CNR10*) and skin color related genes (*Nac078* and *TTG1*) among accessions (Cao *et al.* 2013) in PL, PMC, WK, WD, and WM. (CP = PL + PMC; WP = WK + WD +WM).

Supplementary Tables

Supplementary Table 1 General information of cultivated peaches and their closely relative species distributed in China.

Clade	*Prunus* subg. *Amygdalus*												
Section	*Persica*									*Amygdaluys*	Other closely wild species		
Group	Cultivated peach (CP)			Wild relative peach (WP)						Cultivated almond (CA)			
				WK	WD				WM				
Species	*P. persica* (L.) Batsch		*P. ferganensis* (Kost. et Riab.) Kov. et Kost.	*P. kansuensis* (Rehd.) Kov. et Kost.	*P.davidiana* (Carr.) Franch	*P. davidiana* var. *potanini* (Batalin) Rehder	*P. tangutica* (Batalin) Koehne	*P. mongolica* (Maxim.) Richer	*P. mira* Koehne	*P. dulcis* (Mill.) D.A. Webb	*P. ledebouriana* Schleche	*P. pedunculata* (Pall.) Maxim.	*P. triloba* (Lindl.) *Richer*
Chromosome	2n = 2X = 16 (diploid)		2n = 2X = 16 (diploid)	2n = 2X = 16 (diploid)	2n = 2X = 16 (diploid)	2n = 2X = 16 (diploid)	2n = 2X = 16 (diploid)	2n = 2X = 16 (diploid)	2n = 2X = 16 (diploid)	2n = 2X = 16 (diploid)	2n = 2X = 16 (diploid)	2n = 12X = 96 (dodecaploid)	2n = 8X = 64 (octaploid)
Common Chinese name	Tao		Xinjiang Tao	Gansu Tao	Shan Tao	Shangan Shan Tao	Xikang Bian Tao	Mongolian Bian Tao	Guanghe Tao or Tibet Tao	Bian Tao	Wild Bian Tao or Wild Ba Dan	Changbing Bian Tao	Yü Ye Mei
Type	Cultivars	Landraces	Landraces	Wild species	Wild species	Wild species	Wild species	Wild species	Wild	Landraces	Wild species	Wild species	Wild species
Fruit size	Large	Large	Large	Small	Small	Small	Small	Small	Small	Small	Small	Small	Small
Fruit skin color	More depostion	Less or no depostion	No deposition	No deposition	No deposition	No deposition	No deposition	No deposition	Less or no depostion	No deposition	No deposition	No deposition	More depostion
Fruit mesocarp	Edible: fleshy	Edible: fleshy	Edible: fleshy	Edible: fleshy	Inedible: thin, dry and unsplitting	Inedible: thin, dry and unsplitting	Inedible: thin, dry and splitting	Inedible: thin, dry and splitting	Edible: fleshy	Inedible: thin, dry and splitting	Inedible: thin, dry and splitting	Inedible: thin, dry and splitting	Inedible: thin, dry and splitting
Other main characteristics	Deciduous tree; Fruit phenotypic diversity: fuzz (peach)/fuzz-less (nectarine) fruit, round/flat fruit, white/yellow fruit flesh, clingstone/freestone flesh adhesion, melting/non-melting flesh texture, and acid/non-acid flesh taste		Deciduous tree; Prominent, elongated, unbranched secondary leaf veins roughly parallel to the leaf margin; Parallelly large and deep longitudinal grooves on stone; Tolerance to cold and drought	Deciduous tree; Stones are similar to atypical *P.mira* peaches with deep grooves; Tolerance to cold and drought; Early flowering; Extremely productive	Deciduous shurb or tree; Small pits or very short grooves on stone surface; Short furry fruit; High tolerance to cold, drought, barren and salinity-alkalinity; High resistance to insects and diseases; Earlier flowering than *P. kansuensis*	Deciduous tree; Similar botany characters to *P. davidiana*; Larger and more ellipsoid to ovate than spheroidal stones of *P. davidiana*; Stronger tolerace to drought than *P. davidiana*	Deciduous shrub; Short furry fruit; Stronger tolerance to cold, drought and barren, and resistance to diseases and insects, and spring dry air than *P. davidiana*; Strong adaptability; Longevity and productive	Deciduous shrub; Ancient relict species; Short furry fruit; Productive; Extremely tolerance to drought, cold, barren; Strong adaptability	Deciduous tree; Most with smooth stones and a few with grooves; No pits on stone surface; Longevity and extremely productive; Tolerance to cold and drought	Deciduous tree; Short furry fruit; Edible kernels; Lower nativity and resistance than those of Mediterranean origin; Tolerance to drought; No deep furrows on stones	Deciduous shrub; Relict wild species; Densely furry fruit; High tolerance to cold, drought; Strong adaptability	Deciduous shrub; Densely short furry fruit; Productive; High tolerace to drought, cold and barren; Strong adaptability; High resistance to wind-sand and insects	Deciduous shrub or small tree; Densely short furry fruit; High tolerance to cold, drought, barren and salinity-alkalinity; Strong adaptability
Geographic distribution and habitat	China: widely cultivated in China covering nearly all Chinese provinces except for Heilongjiang		Northwest China: West Tarim Basin of South Xinjiang (Kashga, Hotan and Aksu) and Hexi Corridor of Gansu (Wuwei, Zhangye and Dunhuang); Dry deserts, gobi, and mountain valleys; Altitude: largely 1,000~4,300 m	Northwest China: Gansu, Shaanxi, South Shanxi, West Henan, North Hubei, Northwest Sichuan; Largely in Northwest China including Gansu and Shaanxi; Mountain slopes, valleys, and forest margins; Altitude: largely 600~2,300 m	Northwest and North China: Hebei, Henan, Shanxi, Shandong, Shaanxi, Gansu, Sichuan; Mountain bottoms, slopes, valleys and ridges, and wild thin woodland, and forest; Altitude: mainly 800~3,200 m	Northwest China: West Shaanxi, South Gansu and North Sichuan; Mountain areas resemble to those of *P. davidiana* or *P. kansuensis*; Altitude: 900~2,000 m	Northwest China: South Gansu and Northwest Sichuan; Mountain slopes and valleys; Altitude: 1,500~2,800 m	Northwest China: South Mongolian plateau (Yinshan Mountain), Hexi Corridor of Gansu, Helan Mountain of Ningxia; Desert, gobi, desert steppe, stony slopes and dry valleys; Altitude: 1,000~2,600 m	Southwest China: South and East Tibet, Northwest Yunnan and West Sichuan; Slopes and valleys, and margins of Tibetan Plateau; Altitude: largely 2,500~3,600 m	Northwest China: South Xinjiang, Gansu and Shaanxi; Largely in South Xinjiang; Arid and semi-arid inland with hot, dry and little rain summer and cold winter, mountain regions and Hilly lands; Altitude: 800~1,300 m	Northwest China: North Xinjiang, Mountain slopes or valleys of Barlikhtaw, Tarbagatai and Altai Mountains; Altitude: 900~1,100 m	Northwest China: West Inner Mongolia, Ningxia and North Shaanxi; Semi-arid and arid mountain stone slopes, desert steppes and sand; Altitude: 1,300~1,600 m	Native to Gansu; commonly Northeast, Northwest and North China; Mountain slopes and valleys, and margin of forests and thickets; Altitude: 600~2,500 m
Main use	Economically important tree for edible fruits		Locally important edible fruit tree; Rootstocks for peach	Ecological and ornamental tree; Rootstocks for peach and almond in Northwest China; Breeding materials as early flowering and tolerance to cold and drought; locally edible fruit tree	Rootstocks for stone fruit trees in North China; Ornamental early spring flowering trees; Breeding materials tolerance to cold and drought, and resistance to diseases and insects; Oil and medicinal kernels	Rootstocks for stone fruit trees in Northwest China	Ecological and ornamental tree; Dwarf rootstocks for peach (better than *P. davidiana*) and almond; Breeding materils resistance to diseases and insects; Oil and medicinal kernels	Ecological tree; Oil and medicinal kernels; Rootstone for almond	Rootstocks for peach and almond; Locally edbile fruit for human and livestock (mostly Tibetan pig and yak); Medicinal kernels; Locally important ecological tree	Economically important nut fruit for edible kernels; Rootstock for peach	Ornamental tree; Medicinal kernels; Excellent dwarf rootstocks for stone fruit trees; High oil kernels	Ecological and ornamental tree; Oil and medicinal kernels; Dwarf rootstocks for almond and peach	Ornamental early spring flowering trees (commonly called flowering almond); Dwarf rootstocks for peach and plum; Oil and medicinal kernels
Fruit picture	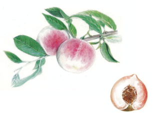		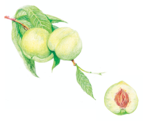	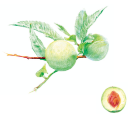	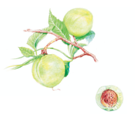	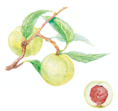	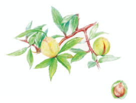	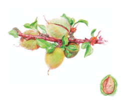	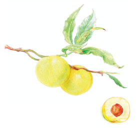	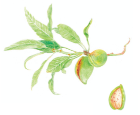	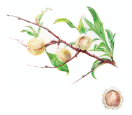	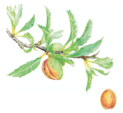	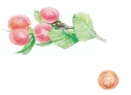

Supplementary Table 2 Sequencing quality of the 44 cultivated peaches and closely relative species sequenced in this study.

Section	Group	Sample code	SRA code	Species	Common name	Origin	Raw bases (bp)	High-quality data				
								Clean data (bp)	Effective rate (%)	Proportion of Q20 (%)	Proportion of Q30 (%)	Proportion of GC (%)
Persica	Cultivated peaches (10)	PL01	SRS1272175	*P. ferganensis*	Kashi 2	Xinjiang, PRC	14,637,205,200	13,499,904,400	92.23	93.35	83.75	38.35
		PL02	SRS1272177	*P. ferganensis*	Kashi You Tao	Xinjiang, PRC	17,492,916,000	16,230,045,800	92.78	93.91	84.78	38.11
		PL03	SRS1272178	*P. ferganensis*	Xinjiang Pan Tao	Xinjiang, PRC	12,963,801,800	12,007,731,200	92.63	93.83	84.65	38.08
		PL04	SRS1272179	*P. ferganensis*	Pan Tao(Weihai)	Shandong, PRC	13,704,460,000	12,676,468,200	92.50	93.62	84.25	38.06
		PL05	SRS1272180	*P. persica*	Mao Tao(Luanchuan)	Henan, PRC	14,371,401,600	13,279,922,800	92.41	93.28	83.53	37.91
		PL06	SRS1272185	*P. persica*	You Tao(Jiyuan)	Henan, PRC	15,822,965,600	14,686,102,800	92.82	93.83	84.60	37.59
		PMC01	SRS1272190	*P. persica*	Sunsplash_1	USA	16,690,751,750	16,452,556,000	98.57	92.75	86.89	38.55
		PMC02	SRS1272192	*P. persica*	Sunsplash_2	USA	15,914,978,200	15,255,370,400	95.86	96.19	89.56	39.33
		PMC03	SRS1272193	*P. persica*	Sunblaze	USA	16,716,918,000	16,446,503,750	98.38	92.42	86.30	39.03
		PMC04	SRS1272194	*P. persica*	Rui Pan 18	Beijing, PRC	14,618,880,000	14,446,430,000	98.82	92.35	86.30	37.89
	Wild relative peaches (12)	WK01	SRS1272209	*P. kansuensis*	Bai Gen Gan Su Tao	Gansu, PRC	15,508,995,000	14,449,758,400	93.17	93.90	84.59	37.97
		WK02	SRS1272211	*P. kansuensis*	Hong Gen Gan Su Tao	Gansu, PRC	14,691,289,200	13,589,577,000	92.50	93.67	84.30	38.05
		WD01	SRS2510354	*P. mongolica*	Meng Gu Bian Tao	Inner Mongolia, PRC	19,730,858,200	18,947,543,800	96.03	95.05	87.12	38.39
		WD02	SRS2510355	*P. tangutica*	Xi Kang Bian Tao	Gansu, PRC	17,069,870,400	16,309,083,000	95.54	95.16	87.46	38.18
		WD03	SRS1272212	*P. davidiana*	Bai Hua Shan Tao	Northern China, PRC	12,745,492,200	11,750,996,000	92.20	93.53	84.15	38.14
		WD04	SRS1273502	*P. davidiana*	Hong Hua Shan Tao	Northern China, PRC	13,242,335,600	12,271,064,400	92.67	93.73	84.36	37.76
		WD06	SRS1273503	*P. davidiana* var. *potaninii*	Shan Gan Shan Tao	Shaanxi, PRC	13,010,170,000	12,002,606,200	92.26	93.32	83.60	37.79
		WM01	SRS1273504	*P. mira*	Guang He Tao	Tibet, PRC	17,344,556,400	16,092,350,600	92.78	93.91	84.75	37.77
		WM02	SRS1270597	*P. mira*	Guang He Tao	Tibet, PRC	20,650,891,500	20,597,688,300	99.74	95.59	90.34	40.24
		WM03	SRS1270602	*P. mira*	Guang He Tao	Tibet, PRC	23,818,603,200	23,732,653,200	99.64	96.88	92.98	41.88
		WM04	SRS1270603	*P. mira*	Guang He Tao	Tibet, PRC	18,307,178,100	18,190,469,400	99.36	95.29	89.83	39.91
		WM05	SRS1270605	*P. mira*	Guang He Tao	Tibet, PRC	15,846,738,300	15,761,688,600	99.46	96.83	92.91	40.11
Amygdalus	Cultivated almonds (15)	DL01	SRS1273505	*P. dulcis*	Shuang Guo	Xinjiang, PRC	14,700,002,750	14,544,182,750	98.94	93.39	87.95	38.46
		DL02	SRS1273506	*P. dulcis*	Zhi Pi	Xinjiang, PRC	13,233,459,250	13,087,891,250	98.90	92.87	87.09	38.10
		DL03	SRS1273507	*P. dulcis*	Gong Ba Dan	Xinjiang, PRC	14,301,125,000	14,053,715,500	98.27	92.87	87.13	38.25
		DL04	SRS1273508	*P. dulcis*	Wan Feng	Xinjiang, PRC	13,945,222,250	13,648,189,000	97.87	92.24	86.04	38.03
		DL05	SRS1273509	*P. dulcis*	Ai Feng	Xinjiang, PRC	13,765,051,000	13,605,376,500	98.84	93.02	87.29	38.50
		DL06	SRS1273510	*P. dulcis*	Ba Dan Wang	Xinjiang, PRC	15,103,937,250	14,915,138,000	98.75	93.00	87.39	38.51
		DL07	SRS1273511	*P. dulcis*	Huang Shuang	Xinjiang, PRC	14,070,239,250	13,774,764,250	97.90	92.05	85.75	38.30
		DL08	SRS1273512	*P. dulcis*	A Yue Hun Zi	Xinjiang, PRC	14,069,751,500	13,846,042,500	98.41	92.47	86.46	38.02
		DL09	SRS1273513	*P. dulcis*	Tao Ba Dan	Xinjiang, PRC	14,611,473,000	14,342,622,000	98.16	92.31	86.22	38.09
		DL10	SRS1273514	*P. dulcis*	Da Ba Dan	Xinjiang, PRC	17,352,546,400	16,314,864,200	94.02	93.94	85.10	39.22
		DL11	SRS1273548	*P. dulcis*	Ye Er Qiang	Xinjiang, PRC	14,460,812,500	14,168,704,000	97.98	92.51	86.59	37.91
		DL12	SRS1273549	*P. dulcis*	Bian Zui He	Xinjiang, PRC	13,654,205,500	13,389,314,000	98.06	91.56	84.84	38.54
		DMC13	SRS1273551	*P. dulcis*	Ao 2	USA	12,515,040,500	12,357,351,000	98.74	92.33	86.19	38.56
		DMC14	SRS1273552	*P. dulcis*	Nonpareil	USA	18,284,527,200	17,346,531,000	94.87	94.36	85.88	38.70
		DMC15	SRS1273553	*P. dulcis*	Mission	USA	13,144,457,250	12,981,466,000	98.76	92.31	85.99	38.64
Other closely wild relatives in China (7)		RW01	SRS2510312	*P. ledebouriana*	Ye Ba Dan	Xinjiang, PRC	17,223,485,100	17,159,758,200	99.63	94.67	88.94	39.84
		RW02	SRS2510317	*P. ledebouriana*	Ye Ba Dan	Xinjiang, PRC	16,420,201,500	16,374,225,000	99.72	94.47	88.55	40.05
		RW03	SRS2510318	*P. ledebouriana*	Ye Ba Dan	Xinjiang, PRC	17,629,865,700	17,569,924,200	99.66	94.39	88.43	39.33
		RW04	SRS2510350	*P. ledebouriana*	Ye Ba Dan	Xinjiang, PRC	22,011,835,200	21,941,397,300	99.68	94.38	88.39	38.86
		RW05	SRS2510353	*P. ledebouriana*	Ye Ba Dan	Xinjiang, PRC	16,078,372,200	16,030,137,000	99.70	94.57	88.67	38.58
		RW06	SRS2510356	*P. pedunculata*	Chang Bing Bian Tao	Shaanxi, PRC	20,196,355,600	19,165,040,000	94.89	94.31	85.78	38.72
		RW07	SRS2510357	*P. triloba*	Yü Ye Mei	Shannxi, PRC	18,213,517,600	17,356,470,200	95.29	94.91	86.98	38.86
				**Total**			699,886,739,750	676,649,618,100	-	-	-	-
				**Average**			-	-	-	93.76	86.65	38.62

Supplementary Table 3 Accuracy estimation of the identified SNPs from our sequencing data.

Sample code	Common name	Accuracy (%)		
		Homozygous loci	Heterozygous loci	Total
PMC01	Sunsplash_1	96.72	98.88	97.36
PMC02	Sunsplash_2	96.73	98.90	97.37
PMC03	Sunblaze	98.00	98.61	98.12
PMC04	Rui Pan 18	97.00	99.08	97.65

Supplementary Table 4 Genetic variation among or within cultivated peaches (CP), wild relative peaches (WP), and cultivated almond (CA)

Group	CP			WP				CA
	PL	PMC	Total	WK	WD	WM	Total	
$\theta\pi$ ($\times10^{-3}$)	1.944	1.9431	2.1524	2.4056	9.6995	2.7782	10.0086	6.611
Tajima's D	0.5218	0.0078	0.1001	-	-	-	-	-

Comparison	WP/CP	WP/PL	PL/PMC	CA/CP	
F_{ST}	0.2498	0.1863	0.1033	0.4378	
$\theta\pi$ Ratio	4.6499	5.1485	1.0004	-	-

Supplementary Table 5 Inbreeding coefficent (*F* value) and regions of homozygosity (ROHs) among *P. persica, P. kansuensis, P. davidiana, P. mira*, and *P. dulcis*

Species		ROHs			F value
		Mean Total Length (Mb)	Mean Number	Mean length (kb)	
P. persica	**Total**	93.95	380.00	247.23	0.3192
	**Landrace (PL)**	128.82	532.00	242.14	0.3873
	**Cultivar (PMC)**	76.51	304.00	251.68	0.1550
P. kansuensis		86.04	498.50	172.61	0.0483
P. davidiana		8.95	58.33	153.47	-0.0043
P. mira		73.32	454.60	161.28	0.1234
P. dulcis		6.84	47.33	144.50	-0.0909

Supplementary Table 6 Statistics and distribution of CNVs for each accession of the 58 high-coverage genomes.

Data set	Group	Sample code	Common name	Upstream	Exonic	Intronic	Downstream	Upstream/ Downstream	Intergenic	Duplication	Deletion	Duplication	Deletion	Total
**58 high-coverage genomes including 44 genomes sequenced in this study and 14 genomes from Verde *et al*. (2013)**	**Cultivated peaches**	PL01	Kashi 2*	359	922	80	279	54	2,254	810	3,304	5,868,400	14,252,900	4,114
		PL02	Kashi You Tao*	356	960	83	306	53	2,298	769	3,462	4,764,100	14,642,600	4,231
		PL03	Xinjiang Pan Tao*	287	812	76	282	44	2,240	895	2,994	6,165,200	13,378,400	3,889
		PL04	Pan Tao(Weihai)*	290	784	70	226	48	2,005	728	2,847	5,123,800	14,866,100	3,575
		PL07	Prunus ferganensis[#]	77	3,018	9	84	12	990	3,394	851	95,427,200	7,588,000	4,245
		PL05	Mao Tao(Luanchuan) *	279	690	64	218	40	1,877	827	2,471	5,506,700	10,988,500	3,298
		PL06	You Tao(Jiyuan)*	336	818	84	276	36	2,334	844	3,200	4,916,200	14,478,800	4,044
		PL08	Sa Hua Hong Pan Tao[#]	188	546	33	153	31	1,563	1,000	1,612	8,663,100	11,420,300	2,612
		PL09	Shen Zhou Mitao	173	553	20	143	19	1,545	1,266	1,248	12,485,100	8,623,700	2,514
		PMC01	Sunsplash_1*	264	600	56	202	48	1,849	704	2,468	4,184,200	9,471,800	3,172
		PMC02	Sunsplash_2*	247	610	53	204	41	1,834	677	2,443	4,141,100	9,051,700	3,120
		PMC03	Sunblaze*	381	761	66	266	79	2,044	696	3,112	4,027,100	14,002,300	3,808
		PMC04	Rui Pan 18*	253	598	66	162	34	1,991	774	2,439	4,549,600	8,800,900	3,213
		PMC05	Oro A[#]	127	491	20	116	23	1,209	735	1,325	7,542,100	10,908,800	2,060
		PMC06	GF305[#]	121	377	28	119	17	1,240	676	1,305	6,786,800	7,453,900	1,981
		PMC07	Bolero[#]	107	363	21	70	15	988	672	941	8,246,700	6,113,700	1,613
		PMC08	F1(Contender x Ambra)[#]	143	391	22	123	31	1,075	745	1,141	8,905,600	6,390,000	1,886
		PMC09	IF7310828[#]	131	302	23	95	19	1,111	585	1,151	6,401,300	6,169,700	1,736
		PMC10	Yumyeong[#]	143	485	31	144	25	1,467	779	1,591	5,673,300	9,931,800	2,370
		PMC11	Quetta[#]	81	216	18	65	12	908	440	907	4,610,000	4,745,200	1,347
		PMC12	Earligold[#]	114	1,057	18	103	15	1,352	1,742	990	14,934,800	5,337,800	2,732
		PMC13	PLov2-2Nc (Reference genome)[#]	227	277	48	167	36	1,527	402	2,013	2,721,600	4,874,800	2,415
	**Wild relative peaches**	WK01	Bai Gen Gan Su Tao*	580	1,637	96	447	101	3,242	974	5,421	5,365,800	31,718,800	6,395
		WK02	Hong Gen Gan Su Tao*	561	1,647	103	445	104	3,284	1,057	5,372	6,017,000	29,609,100	6,429
		WK03	*Prunus kansuensis* (Clone P1429)[#]	207	1,433	15	164	26	1,478	1,154	2,317	16,706,500	22,097,100	3,471
		WD01	Meng Gu Bian Tao*	603	1,599	60	406	103	2,576	848	4,800	4,575,900	36,546,400	5,648
		WD02	Xi Kang Bian Tao*	689	1,871	84	523	107	3,483	956	6,158	4,907,600	36,725,100	7,114
		WD03	Bai Hua Shan Tao*	558	1,602	73	400	108	2,561	940	4,610	6,305,300	34,682,900	5,550
		WD04	Hong Hua Shan Tao*	602	1,700	78	440	108	2,948	994	5,179	6,581,900	36,334,200	6,173
		WD05	*Prunus davidiana* (Clone P 1908)[#]	297	1,503	29	245	57	1,778	1,110	3,022	11,486,300	28,029,800	4,132
		WD06	Shan Gan Shan Tao*	556	1,586	62	402	108	2,583	955	4,626	5,892,600	33,234,100	5,581
		WM01	Guang He Tao*	794	1,823	106	533	129	3,513	978	6,303	5,367,700	36,653,700	7,281
		WM02	Guang He Tao*	775	1,839	108	533	137	4,032	964	6,863	4,961,800	39,552,400	7,827
		WM03	Guang He Tao*	768	1,810	103	535	127	4,088	970	6,873	5,167,500	39,219,700	7,843
		WM04	Guang He Tao*	376	2,196	26	256	74	2,258	1,790	3,660	25,876,200	28,885,400	5,450
		WM05	Guang He Tao*	677	1,697	84	442	109	3,550	971	5,932	5,553,400	36,034,900	6,903
	**Cultivated almonds**	DL01	Shuang Guo*	627	1,846	92	406	114	3,255	966	5,670	5,240,900	35,880,900	6,636
		DL02	Zhi Pi*	546	1,809	71	387	97	3,029	993	5,214	5,829,600	34,113,700	6,207
		DL03	Gong Ba Dan*	567	1,862	75	397	105	3,023	984	5,331	5,707,500	34,813,100	6,315
		DL04	Wan Feng*	580	1,840	89	418	102	3,001	1,017	5,301	5,801,700	34,674,300	6,318
		DL05	Ai Feng*	546	1,854	83	419	108	2,933	955	5,277	5,721,300	35,541,200	6,232
		DL06	Ba Dan Wang*	660	1,831	84	432	112	3,257	942	5,708	5,272,700	35,604,900	6,650
		DL07	Huang Shuang*	486	1,801	66	373	89	2,508	950	4,621	6,029,100	33,242,600	5,571
		DL08	A Yue Hun Zi*	575	1,817	63	400	102	2,921	937	5,195	5,663,200	33,906,500	6,132
		DL09	Tao Ba Dan*	586	1,888	67	402	101	2,911	974	5,270	5,529,400	35,553,300	6,244
		DL10	Da Ba Dan*	479	1,707	48	314	82	2,239	948	4,155	6,715,200	32,440,100	5,103
		DL11	Ye Er Qiang*	623	1,941	94	410	109	3,413	1,025	5,871	5,654,500	36,061,200	6,896
		DL12	Bian Zui He*	432	1,736	45	321	88	2,239	994	4,129	6,800,200	32,213,200	5,123
		DMC13	Ao 2*	452	1,694	48	306	90	2,132	945	4,012	6,743,000	31,662,200	4,957
		DMC14	Nonpareil*	526	1,734	72	354	94	2,409	955	4,498	5,929,800	32,881,100	5,453
		DMC15	Mission*	441	1,685	56	326	88	2,322	942	4,240	6,559,300	32,143,500	5,182
	**Other wild relatives**	RW01	Ye Ba Dan*	410	1,747	43	257	93	1,421	494	3,790	4,627,900	47,347,000	4,284
		RW02	Ye Ba Dan*	445	1,812	41	242	106	1,463	546	3,921	5,715,100	47,363,400	4,467
		RW03	Ye Ba Dan*	433	1,794	57	282	104	1,582	474	4,107	3,066,500	49,783,900	4,581
		RW04	Ye Ba Dan*	481	1,811	47	280	95	1,646	495	4,181	3,169,300	51,117,300	4,676
		RW05	Ye Ba Dan*	524	1,894	65	335	135	1,680	464	4,534	2,412,900	54,727,700	4,998

The samples with >10× sequencing depth were markered with * and [#] were used for subsequent analyses

* Raw data were genetated in this study

[#] Raw data were downloaded from Verde *et al*. 2013

Supplementary Notes

Supplementary Note 1 Phylogenetic analysis of the 58 samples along with *P. mume*

A phylogenetic analysis based on the 58 samples along with *P. mume* (belonging to the genus *Prunus* subgenus *Prunus*) placed *P. ledebouriana, P. triloba*, *P. pedunculata*, and *P. mume* into separate clades from the well-established monophyletic *Prunus* subg. *Amygdalus* clade (Supplementary Fig. 2). This is evident on the basis of genome-wide SNPs strongly support the previous taxonomy and classification which addressed that *P. triloba* and *P. pedunculata* should be excluded from subgenus *Amygdalus* mainly on the basis of chloroplast DNA sequences[1], although *P. pedunculata* and *P. trilob*, both of which belong to a sister clade of the peach (*Persica* section) clade in phylogenetic analysis of morphological data[1]. Despite the fact that small shrub *P. ledebouriana* (previously named 'wild almond', endemic to the valleys of Barlikhtaw, Tarbagatai and Altai Mountains of north to Tian Shan Mountains in central Asia, northern Xinjiang, northwest China) shares a close geographic distribution with cultivated almond *P. dulcis* which mainly resides in southwest to Tian Shan Mountains, southern Xinjiang, these two species showed no closely affinity as was previously thought. Therefore, *P. triloba*, *P. pedunculata*, along with *P. mume* were not included in subsequent analyses, whereas *P. ledebouriana* was used as the outgroup for the analyses of population structure in *Prunus* subg. *Amygdalus* species.

Supplementary Note 2 An increased inbreeding level in edible peach species associated with selection for favorite fruit traits

It is now understood that there is no absolute self-compatible (SC) or self-incompatible (SI) peach species, even cultivated peach (*P. persica*), defined as SC species, has cross-pollinator cultivars owing to male sterility[2]. Hence, it is more accurate to state that the SI peach species like *P. mongolica*, *P. tangutica*, and *P. davidiana* hold much lower inbreeding levels compared to that of *P. mira*, and *P. kansuensis*, and *P. persica* which considered as a SC species shows the highest inbreeding level among these peach species (supported by our analyses of the extent of ROHs, F-value, and heterozygosity results). As SI is predominant in *Prunus* and SC is derived from diverse types of dysfunction mutants in S-locus (also including the natural mutations associated with transposable elements (TEs) insertion[3]) of these *Prunus* species, we considered that the population of SC was increased (reflecting on the increasing of inbreeding) when selection was exerted on these three edible peach species: it is conceivable that frugivores could exert selection based on fruit characters but not the mating system of peach species. However, maintaining favorable fruit traits have been strongly impacted by the higher inbreeding levels of these peach species. Thus, these two domestication syndromes (inbreeding level and improved fruit edibility) were related during the evolution of peach species.

Supplementary Note 3 Granger causality test for assessing N_e trend similarity between monkey and peach species

The Granger causality (GC) test was applied to assess if there is a similar trend in the effective population size (N_e) between the three different monkey species and the five peach species across different age periods. GC test is a statistical hypothesis test commonly applied in time series analysis aimed to determine whether one time series is capable in predicting another, first proposed by Granger (1969)[4]. In our application, we tried to test if the monkey N_e can predict the N_e change in *P. dulcis* and the peach species (*P. mira*, *P. davidiana*, *P. kansuensis*, and *P. persica*). The significant test results implied that the N_e of the each of the southwest monkey species (*Rhinopithecus brelichi* and *R. bieti*) can be used to predict the N_e trajectories of the three edible peach species (*P. mira*, *P. kansuensis*, and *P. persica*). The GC test was performed using the grangertest() function implemented in the lmtest package in R.

Table *P*-value for testing the trend similarity between monkey and peach species

Monkey species	Time range (Thousand years ago)	*P. dulcis*	*P. mira*	*P. davidiana*	*P. kansuensis*	*P. persica*
R. brelichi	8–900	0.33	0.005**	0.368	0.003**	0.0029**
R. bieti	8–900	0.23	0.017*	0.50	0.004**	0.002**
R. strykeri	8–500	0.276	0.051	0.92	0.133	0.101

(*: $P \leq 0.05$; **: $P \leq 0.01$)

Supplementary Note 4 Phylogenetic relationship and evolved edible mesocarp in *Persica* section species

Two phylogenetic tree based on the data from Yazbek *et al*., 2014 (**a**) and the result from our study (**b**) are shown below. The assumed common ancestors of *Amgdalus* and *Persica* sections' species are SI and bears dry, splitting inedible mesocarps[5,6], and the transition from inedible mesocarps to edible mesocarps occurred in *Persica* section species. Two subclades, including inedible- and edible-mesocarp species are shown on the phylogenetic tree (**a**), and *P. davidiana* represents as a transition between peaches species with fleshy-non-splitting and dry-splitting mesocarp[5,6]. Our result suggested that *P. mira* was the earliest diverged wild relative species in *Persica* section, and the evolved edible mesocarp in *P. mira* likely contributed by the introgression from the common ancestor of *P. kansuensis* and *P. persica*.

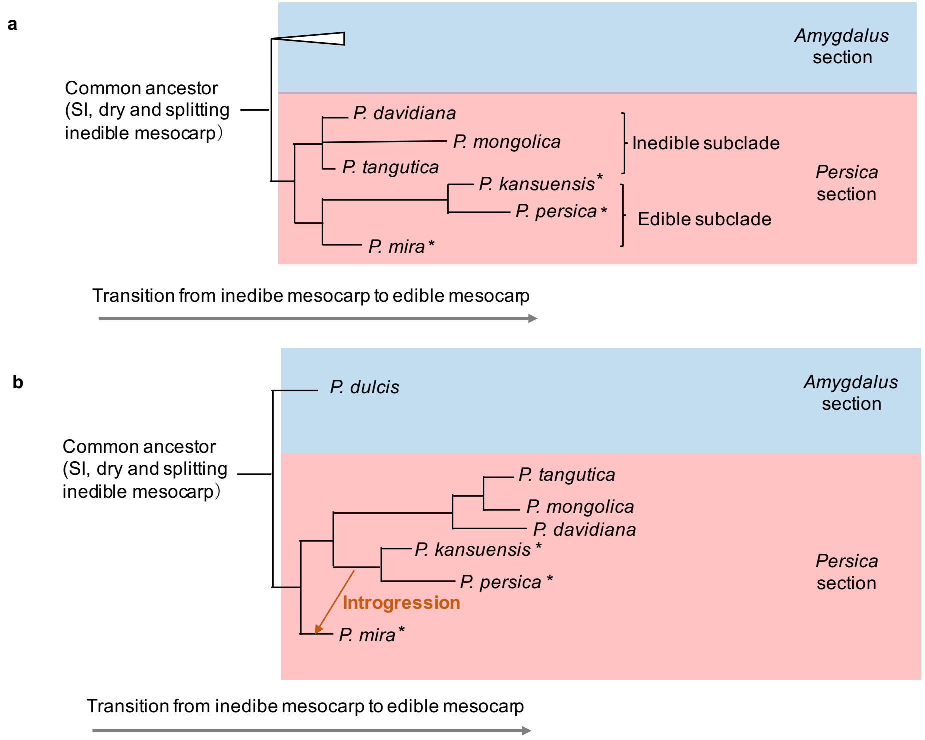

Supplementary Note 5 Maintenance of favorable traits in self-incompatible (cross-pollinated) species is harder than in self-compatible (inbreeding) species

The increased diversity and heterozygosity of self-incompatible (cross-pollinated) species may bring evolutionary advantage with increased survival probability of their progeny in changing environmental or stress conditions, in this case *P. mongolica*, *P. tangutica*, and *P. davidiana* in *Persica* section. However, under long-term open pollination conditions, high heterozygosity will also impair the maintenance of favorable traits, leading to phenotypic differentiation, thus hindered the evolution of fruit traits.

Supplementary Note 6 *P. mira* and *P. kansuenisis* are staying at pre-domestication stage

In addition to *P. persica,* there are two other peach species bearing edible fleshy mesocarps: *P. mira* (also known as "Tibetan peach") and *P. kansuensis* (also named as "juicy peach" in Shaanxi, Gansu, and Shanxi province). These two wild relatives are mostly distributed in natural forests, and fruit is occasionally gathered by humans, yet without intentional selection. It has been proposed that the definition of plant domestication should refer to more concrete, positive human intervention[7]. In this process, changes in cultivation habitat (adaptation) and choosing individuals with favorable traits for cultivation (selection) are critical. However, fruit gathering from a natural forest would not be considered as a domestication process with such assumptions (like the scenario of kiwifruit whose edible fruit has been occasionally collected in western China for human consumption, while the actual adaptation and cultivation of kiwifruit took place only during the twentieth century in New Zealand[8]).

A "home garden" cultivation for perennial fruit trees is also distinct from the domestication of grain and pulse crops, since a relatively short-distance shifting of habitat and a weak selection occurred due to very a limited number of cultivation individuals (even a single tree could provide food for whole family), and the long generation time. This supports a statement that these kind of species, in this case *P. mira* and *P. kansuenisis*, may have experienced a selection process (likely mediated by frugivores) but still did not undergo a domestication mediated by humans, representing a fascinating pre-domestication stage.

References

1. Yazbek, M. & Oh, S.H. Peaches and almonds: phylogeny of *Prunus* subg. *Amygdalus* (Rosaceae) based on DNA sequences and morphology. *Plant Syst. Evol.* **299**, 1403–1418 (2013).
2. Loreti, F. & Morini. S. Propogation techniques. In: Layne DR, Bassi D, editors. The Peach: Botany, Production and Uses.Wallingford: CABI. 221–243 (2008).
3. Halász, J., Kodad, O. & Hegedűs, A. Identification of a recently active *Prunus*-specific non-autonomous Mutator element with considerable genome shaping force. *Plant J.* **79**, 220–231(2014).
4. Granger, C. W. Investigating Causal Relations by Econometric Models and Cross-spectral Methods. *Econometrica* **37**, 424–438 (1969).
5. Yazbek, M. & Oh, S.H. Peaches and almonds: phylogeny of *Prunus* subg. *Amygdalus* (Rosaceae) based on DNA sequences and morphology. *Plant Syst. Evol.* **299**, 1403–1418 (2013).
6. Yazbek, M.M. & Al-Zein, M.S. Wild almonds gone wild: revisiting Darwin's statement on the origin of peaches. *Genet. Resour. Crop Evol.* **61**, 1319–1328 (2014).
7. Janick, J. The origin of fruits, fruit growing and fruit breeding. Plant Breeding Reviews **25**, 255–320 (2005).
8. Huang, H. W. & A. R. Ferguson. Genetic resources of kiwifruit domestication and breeding. Horticultural Reviews **33**,1–121 (2007).
