# Supplementary material for: Genome re-sequencing reveals the evolutionary history of peach fruit edibility
Source: Nat Commun. 2018 Dec 20;9:5404. doi: 10.1038/s41467-018-07744-3 (PMC6302090; doi:10.1038/s41467-018-07744-3)
Supplement: Supplementary file 3 — Description of Additional Supplementary Files [file 41467_2018_7744_MOESM3_ESM.pdf]

Description of Additional Supplementary Files

Supplementary Data 1 Genome mapping and coverage information for all accessions analyzed in this study.

Supplementary Data 2 Distribution of SNPs in different genomic regions for each accession.

Supplementary Data 3 SNP loci for validation using a customized Illumina peach array.

Supplementary Data 4 Introgressed segments between *P. mira* and *P. kansuensis*, and between *P. mira* and *P. persica*.

Supplementary Data 6 GO enrichment of predicted genes in introgressed segments (Significant (*P*-value < 0.05) enriched terms).

Supplementary Data 7 Genes putatively related to fruit edibility in introgressed segments.

Supplementary Data 8 Putative sweep regions (Comparison groups: WP/CP, WP/PL, and PL/PMC).

Supplementary Data 9 Genes within the putative sweep regions.

Supplementary Data 10 Significant enriched GO terms among predicted genes in sweep regions.

Supplementary Data 11 Candidate genes related to fruit edibility within sweep regions.

Supplementary Data 12 Significantly enriched GO terms that contain candidate genes related to fruit edibility.

Supplementary Data 13 Putatively selected V_{ST} regions (Top 5%) (Comparison groups: WP/CP, WP/PL, and PL/PMC).

Supplementary Data 14 Genes within the top 5% V_{ST} regions.

Supplementary Data 15 Significant enriched GO terms (*P*-value < 0.05) among predicted genes in top 5% V_{ST} regions.

Supplementary Data 16 Candidate genes related to fruit edibility within top 5% V_{ST} regions.

Supplementary Data 17 Candidate genes related to fruit size.

Supplementary Data 18 Candidate genes related to fruit skin color.
